# Capacity of a Radio Vortex Communication System Using a Partial Angular Aperture Receiving Scheme under the Horizontal Non-Kolmogorov Model

**DOI:** 10.3390/s21051778

**Published:** 2021-03-04

**Authors:** Qian Ma, Hengkai Zhao

**Affiliations:** School of Communication and Information Engineering, Shanghai University, Shanghai 200444, China; maqian@shu.edu.cn

**Keywords:** radio vortex (RV) communication system, partial angular aperture receiving (PAAR) scheme, horizontal non-Kolmogorov channel model, channel capacity

## Abstract

A partial receiving scheme based on limited angular aperture multi-beam receiving and demultiplexing can solve the difficulty caused by the divergence of the vortex beam in the conventional whole beam receiving scheme and realize the long-distance transmission of the vortex wave. The propagation of the radio vortex beam in atmospheric turbulence is of significant importance in theoretical study and practical applications. In this paper, the influence of atmospheric turbulence on the performance of a radio vortex (RV) communication system based on a partial angular aperture receiving (PAAR) scheme under the horizontal non-Kolmogorov channel model is studied. The spiral spectrum of the PAAR scheme and the channel capacity of the RV communication system using the PAAR scheme are derived. Simulation results demonstrate that the selected transmission frequency range has a great influence on the RV communication system based on the PAAR scheme, and the choice of the orbital angular momentum (OAM) mode number L has an influence on the propagation distance. The capacity of RV communication systems based on the PAAR scheme increases with the increase of the transmission frequency in the selected transmission frequency range of 10 GHz–60 GHz. When the number of orbital angular momentum (OAM) modes L is small, we can improve the signal-to-noise ratio (SNR) to obtain a larger capacity of the RV communication system based on the PAAR scheme over a longer propagation distance.

## 1. Introduction

Orbital angular momentum (OAM) has attracted much interest due to its intrinsic physical properties and has been extensively studied in the field of wireless communications [1,2,3]. Experimental and simulated demonstrations have also validated that OAM technology can provide a new degree of freedom for wireless communication systems, which means that when OAM technology is used in 5G wireless communication systems, there exists a potentially revolutionary improvement in wireless capabilities [4].

However, due to the divergence of the OAM beam, the receiver of the OAM-based wireless communication system requires a large receiving aperture in a long-distance link, which is not feasible in practice. In recent years, in order to solve the problem of a too large receiving aperture in a long-distance link, research on partial aperture receiving technology has been done. In [5], a novel partial angular aperture receiving (PAAR) scheme, using a restricted angular aperture to receive and demultiplex multi-OAM mode beams, was proposed, which was carried out to verify the feasibility by experiments. An improved receiving scheme called partial aperture sampling receiving (PASR) was proposed in [6], whose simulated results give a helpful guide on the manipulation of practical OAM-based wireless communication systems. In [7], the influence of n additive white Gaussian noise (AWGN) channel on the PASR scheme was analyzed. The influence of the AWGN channel and the Rician channel on the PASR scheme and the effect of the non-ideal receiving condition on the partially receiving aperture scheme were also analyzed. In [8], the authors used a discretely sampled partial aperture receiver (DSPAR) and showed algebraically that such systems can achieve ideal mode orthogonality in cases where existing partial aperture receivers cannot. In [9], we demonstrated that in the Kolmogorov channel model, the radio vortex (RV) communication system using the PAAR scheme is more stable in the strong turbulence environment than the RV communication system using the whole angular aperture receiving scheme.

The RV communication system using partial aperture receiving technology in the Kolmogorov channel model was analyzed in previous works. However, some experimental results [10,11,12,13,14,15], which showed that atmospheric turbulence may not always obey Kolmogorov’s law, have been published. Motivated by this, we study the RV communication system using the partial aperture receiving scheme in horizontal non-Kolmogorov turbulence (the differences between the two turbulence models are detailed in Appendix A). In this article, the capacity model of the RV communication system using the partial aperture receiving scheme based on the horizontal non-Kolmogorov turbulence model is proposed. It is necessary to transmit multiple modes simultaneously in this paper. However, the number of transmission modes in the scheme of [7] was limited. Therefore, we chose the scheme of [5] to study the influence of atmospheric turbulence on the RV communication system using the partial aperture receiving scheme in the horizontal non-Kolmogorov turbulence model. The remainder of this paper is organized as follows. In Section 2, we propose a channel capacity model of the PAAR scheme based on the non-Kolmogorov turbulence model. In Section 3, the numerical simulation results are analyzed and discussed. Finally, the conclusions are drawn in Section 4.

## 2. System Model and Theoretical Principle

**Figure 1:**
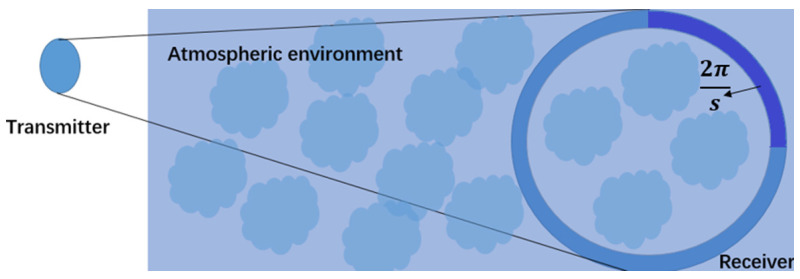
RV communication system using a PAAR scheme in the atmospheric turbulent environment.

In this section, we consider the RV communication system using a PAAR scheme in a horizontal non-Kolmogorov turbulence environment, as shown in Figure 1. For the PAAR scheme, the receiver uses an angular aperture of $\frac{2\pi }{s}$. The Laguerre-Gaussian (LG) beam is used to describe the vortex wave carrying OAM in this paper. In cylindrical coordinates, the functional form of the LG mode in the z plane can be described as [16]:

(1) $${LG}_{p,l_{0}}\left(r,\phi ,z\right)=\kappa \sqrt{\frac{p!}{\pi (p+\left|l_{0}\right|)!}}\frac{1}{\omega (z)}{\left(\frac{\sqrt{2}r}{\omega (z)}\right)}^{\left|l_{0}\right|}e^{-{\left(\frac{r}{\omega (z)}\right)}^{2}}\times L_{p}^{\left|l_{0}\right|}\left(\frac{2r^{2}}{\omega^{2}\left(z\right)}\right)e^{\frac{-i\pi r^{2}}{\lambda R(z)}}e^{i\left(\left|l_{0}\right|+2p+1\right)\Phi \left(z\right)}e^{-il_{0}\phi }$$

where *r* is the radial distance, ϕ is the azimuthal angle, z is the propagation distance, κ is an arbitrary complex constant, *i* is an imaginary unit, *p* is the order of the Laguerre polynomial $L_{p}^{\left|l_{0}\right|}\left(x\right)$, and *p* = 0 is generally configured for RV systems. $L_{p}^{\left|l_{0}\right|}\left(x\right)$ = 1, when p=0. $l_{0}$ is the OAM state transmitted at the transmitter, whose absolute value describes the number of twists of the helical wavefront. $\omega \left(z\right)=\omega_{0}\sqrt{1+{\left(\frac{z}{z_{R}}\right)}^{2}}$, where ω(z) is the beam waist radius of the radio vortex wave at the propagation distance z, $\omega_{0}$ is the beam waist radius of the LG beam at z= 0, and $z_{R}=\frac{\pi \omega_{0}^{2}}{\lambda }$ is the Rayleigh distance. $R\left(z\right)=z\left[1+{\left(\frac{\pi \omega_{0}^{2}}{\lambda z}\right)}^{2}\right]$, where λ is the wavelength. $\Phi \left(z\right)=\arctan\left(\frac{z}{z_{R}}\right)$ is the Gouy phase.

Under the Rytov approximation, when passing through a weak turbulent atmosphere, the beam field received at the receiving aperture at distance *z* is expressed as [17,18]:

(2) $$\begin{array}{c} LG_{p,l}^{\prime }\left(r,\phi ,z\right)={LG}_{p,l_{0}}\left(r,\phi ,z\right)e^{\psi (r,\phi ,z)} \end{array}$$

where ψ(r,ϕ,z) is the phase distortion term caused by the atmospheric turbulence. Based on the quadratic approximation [19], ψ(r,ϕ,z) satisfies:

(3) $$\begin{array}{c} \langle e^{\left(\psi \left(r,\phi ,z\right)+\psi^{*}\left(r,\phi^{\prime },z\right)\right)}\rangle =e^{\frac{2r^{2}}{\rho_{0}^{2}}\left(cos\left(\phi^{\prime }-\phi \right)-1\right)} \end{array}$$

where $\rho_{0}$ is the spatial coherence radius of a spherical wave under the horizontal non-Kolmogorov turbulence model [20]. Based on the mask model proposed in [9], the field received by the partial aperture is expressed as (details are in Appendix B):

(4) $$LG_{p,l}^{P}\left(r,\phi ,z\right)=\frac{1}{s}LG_{p,l}^{\prime }\left(r,\phi ,z\right).$$

According to [21], the field distribution of Equation (4) is expanded according to the spiral spectrum harmonics:

(5) $$LG_{p,l}^{P}\left(r,\phi ,z\right)=\frac{1}{\sqrt{2\pi }}\sum_{l=-\infty }^{\infty }\beta_{l}^{P}\left(r,z\right)e^{-il\phi }$$

where $\beta_{l}^{P}\left(r,z\right)$ is given by the integral formula:

(6) $$\beta_{l}^{P}\left(r,z\right)=\frac{1}{\sqrt{2\pi }}\int_{0}^{2\pi }LG_{p,l}^{P}\left(r,\phi ,z\right)e^{il\phi }d\phi .$$

Therefore, the expression for $\left|\beta_{l}^{P}\left(r,z\right)\right|$ is:

(7) $${\left|\beta_{l}^{P}\left(r,z\right)\right|}^{2}=\frac{1}{2\pi }\int_{0}^{2\pi }\int_{0}^{2\pi }LG_{p,l}^{P}\left(r,\phi ,z\right)LG_{p,l}^{P*}\left(r,\phi ,z\right)e^{il(\phi -\phi^{\prime })}d\phi d\phi^{\prime }.$$

Substituting Equation (4) into Equation (7), then averaging over the turbulence ensembles, and finally, combining Equation (3), we can obtain the spiral mode probability distribution:

(8) $$\langle {\left|\beta_{l}^{P}\left(r,z\right)\right|}^{2}\rangle =\frac{1}{2\pi s^{2}}\int_{0}^{2\pi }\int_{0}^{2\pi }LG_{p,l}^{\prime }\left(r,\phi ,z\right)LG_{p,l}^{\prime *}\left(r,\phi ,z\right)e^{il(\phi -\phi^{\prime })}d\phi d\phi^{\prime }\times e^{-\left(2r^{2}-2r^{2}cos\left(\phi^{\prime }-\phi \right)\right)/\rho_{0}^{2}}$$

where $\rho_{0}$ is the spatial coherence radius of a spherical wave for horizontal atmospheric propagation, given by [20]:

(9) $$\begin{array}{ccc} & \rho_{0}={\left\{\frac{\left(\alpha -1\right)2\Gamma \left(\frac{3-\alpha }{2}\right){\left[\frac{8}{\alpha -2}\Gamma \left(\frac{2}{\alpha -2}\right)\right]}^{(\alpha -2)/2}}{\pi^{\frac{1}{2}}k^{2}\Gamma \left(\frac{2-\alpha }{2}\right){\tilde{C}}_{n}^{2}L}\right\}}^{\frac{1}{\alpha -2}},\; & 3<\alpha <4 \end{array}$$

where α is the non-Kolmogorov turbulence parameter and Γ(x) is the Gamma function. $k=\frac{2\pi }{\lambda }$ is the wave number. ${\tilde{C}}_{n}^{2}$ is a constant with units of m${}^{3-\alpha }$ over the path for horizontal atmospheric propagation [20].

By substituting Equation (1) into Equation (8) and utilizing the integral formula [22] $\int_{0}^{2\pi }e^{-im\phi +\eta cos(\phi -\phi^{\prime })}d\phi =2\pi e^{-im\phi^{\prime }}I_{m}\left(\eta \right)$, we can obtain the final expression of the spiral mode probability distribution of LG beams:

(10) $$\begin{array}{c} \langle {\left|\beta_{l}^{P}\left(r,z\right)\right|}^{2}\rangle =\frac{2\kappa^{2}p!}{s^{2}\left(p+\left|l_{0}\right|\right)!\omega^{2}\left(z\right)}{\left(\frac{2r^{2}}{\omega^{2}\left(z\right)}\right)}^{\left|l_{0}\right|}e^{-\frac{2r^{2}}{\omega^{2}\left(z\right)}-\frac{2r^{2}}{\rho_{0}^{2}}}{\left[L_{p}^{\left|l_{0}\right|}\left(\frac{2r^{2}}{\omega^{2}\left(z\right)}\right)\right]}^{2}I_{l-l_{0}}\left(\frac{2r^{2}}{\rho_{0}^{2}}\right) \end{array}$$

where $I_{l-l_{0}}\left(.\right)$ is the modified $\left(l-l_{0}\right)$-order Bessel function of the first kind.

In PAAR scheme, the weight of any beam’s spiral harmonic *l* is given by

(11) $$\begin{array}{cc} \; & \zeta_{l}^{P}\left(z\right)=\frac{\chi_{l}^{P}\left(z\right)}{\sum_{m=-\infty }^{\infty }\chi_{m}^{P}\left(z\right)} \end{array}$$

which is also known as the spiral spectrum, where $\chi_{m}^{P}$ is given by the integral formula:

(12) $$\chi_{m}^{P}=\int_{0}^{R_{1}}\langle {\left|\beta_{m}^{P}\left(r,z\right)\right|}^{2}\rangle rdr$$

where $R_{1}$ is the receiver radius. It is well known that the radius of the OAM annular region with the maximum energy strength is denoted by [4]:

(13) $$r_{\max}\left(z\right)=\sqrt{\frac{\left|l\right|}{2}}w\left(z\right).$$

$r_{\max}\left(z\right)$ determines the size of the receiver radius. Only when the receiver radius $R_{1}$ is larger than $r_{\max}\left(z\right)$ can the complete energy of the vortex beam be received.

According to [21], $\zeta_{l_{0}}^{P}\left(z\right)$ is defined as the detection probability of the desired OAM mode $l=l_{0}$, which represents the transfer rate of the transmitted OAM state; for $l=l_{0}\pm \Delta l$, $\zeta_{l}^{P}\left(z\right)$ is defined as the crosstalk probability, which denotes the probability that a photon changes its OAM state, and Δl denotes the OAM quantum number difference. For the RV communication system based on the LG beam carrying multiple topological charges, we assume the transmission model is the binary symmetric channel, giving the capacity of the $l_{0}$-th OAM channel in the RV communication system using the PAAR scheme [23]:

(14) $$\begin{array}{cc} \; & C\left(p_{l_{0}}^{P}\right)=1+p_{l_{0}}^{P}\log_{2}\left(p_{l_{0}}^{P}\right)+\left(1-p_{l_{0}}^{P}\right)\times log_{2}\left(1-p_{l_{0}}^{P}\right) \end{array}$$

with flip probability $p_{l_{0}}^{P}$:

(15) $$p_{l_{0}}^{P}=\frac{1}{2}erfc\left(\sqrt{\frac{\gamma^{P}}{2}}\right)$$

where erfc(.) is the complementary error function, $\gamma^{P}$ is the signal-to-interference-plus-noise ratio (SINR) of the $l_{0}$-th OAM channel given by:

(16) $$\gamma^{P}≜\frac{\eta_{l_{0}l_{0}}^{2}}{\sum_{l\in B}^{l\neq l_{0}}\eta_{l_{0}l}^{2}+N_{0}/P_{Tx}\;}$$

where *B* is a transmitted OAM state set. In order to obtain the orthogonality of the partial receiving scheme, the transmitted OAM state set *B* must satisfy orthogonality conditions $l_{n}=l_{1}+ns$ and can be used for multiplexing due to the inherent low crosstalk [5]. $\eta_{l_{0}l}$ is the crosstalk, where $l_{0},l$ means the transmitted OAM channel and the observed OAM channel at the receiver, respectively. $N_{0}$ is the additive white Gaussian noise power. $P_{TX}$ is the transmitted power. Therefore, the capacity of the RV communication system using PAAR under horizontal the non-Kolmogorov turbulence model is:

(17) $$C^{P}=\sum_{l_{0}\in B}C(p_{l_{0}}^{P})$$

## 3. Numerical Results

In this section, we analyze the influence of atmospheric turbulence on the RV communication system using the PAAR scheme in the horizontal non-Kolmogorov turbulence model and discuss factors affecting the channel capacity, including non-Kolmogorov turbulence parameter α, the SNR, structure constant ${\tilde{C}}_{n}^{2}$, transmission frequency *f*, propagation distance *z*, and beam waist radius $w_{0}$. First, we analyze the influence of non-Kolmogorov turbulence parameters α on the channel capacity based on the PAAR scheme. Then, we focus on evaluating the influence of the working frequency *f* and propagation distance *z* on the channel capacity based on the PAAR scheme. The RV communication system using the PAAR scheme has L = 2n + 1 symmetrically distributed OAM states. The default values of the simulation parameters are set as follows: working frequency f=30 GHz, circular arc s=4, radius of the receiver $R_{1}=120$ m, structure constant ${\tilde{C}}_{n}^{2}=1.2\times 10^{-16}$m${}^{3-\alpha }$, signal-to-noise ratio SNR=10 dB, non-Kolmogorov turbulence parameter α=3.97, beam waist radius $w_{0}=0.01$ m, and propagation distance z=80 m. In [24], the vortex phase properties of OAM were well retained after long-distance transmission, which was experimentally demonstrated. Hence, as long as the OAM receiving antenna is improved and the OAM modes of the PAAR scheme satisfy the orthogonality conditions $l_{n}=l_{1}+ns$, it can provide ideal orthogonality for a set of regular OAM modes after long-distance transmission.

**Figure 2:**
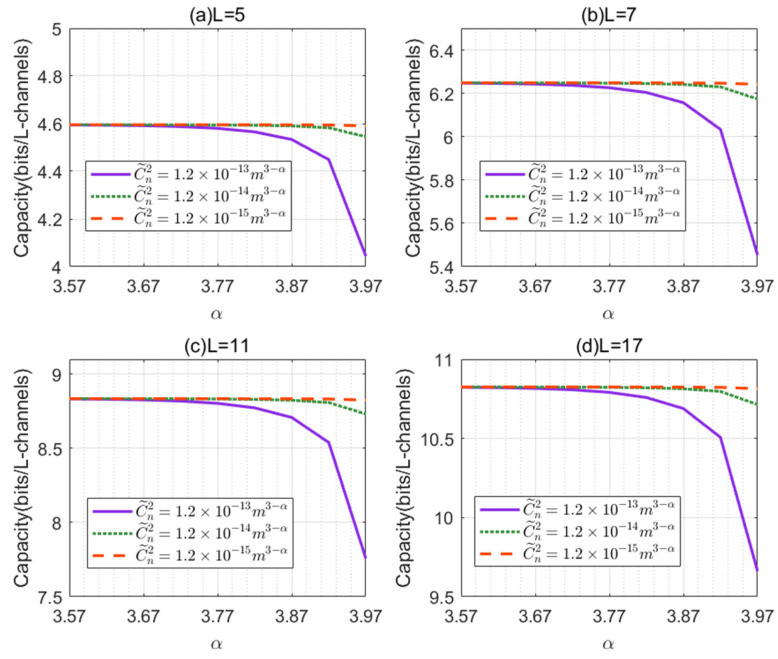
The channel capacity versus the non-Kolmogorov turbulence parameter α for different numbers of orbital angular momentum (OAM) states L: (**a**) L = 5; (**b**) L = 7; (**c**) L=11; (**d**) L = 17.

Figure 2 shows the channel capacity versus the non-Kolmogorov turbulence parameter α for different numbers of OAM states L. We show the difference between the ideal value of the channel capacity and the actual maximum value of the channel capacity, and compare it with the ideal value of the channel capacity. The ratio is called the capacity attenuation. As illustrated in Figure 2, we can see that when L = 5, 7, 11, and 17 (the ideal values of channel capacity are 5, 7, 11, and 17, respectively), the capacity attenuations are about 8.0%, 10.7%, 19.6%, and 36.5%, respectively. We conclude that the larger the number of OAM states L is, the more serious the attenuation of turbulence on the channel capacity is. In Figure 2a–d, we can observe that with identical structure constants (${\tilde{C}}_{n}=1.2\times 10^{-13}$m${}^{3-\alpha }$, $1.2\times 10^{-14}$m${}^{3-\alpha }$, $1.2\times 10^{-15}$m${}^{3-\alpha }$), the channel capacity first is fixed and then gradually decreased as non-Kolmogorov turbulence parameter α increases. We can also observe that the channel capacities first are the same for different structure constants. When turbulence parameter α increases to 3.67, the channel capacity is decreased as structure constants increase under the same turbulence parameter α (α >= 3.67).

**Figure 3:**
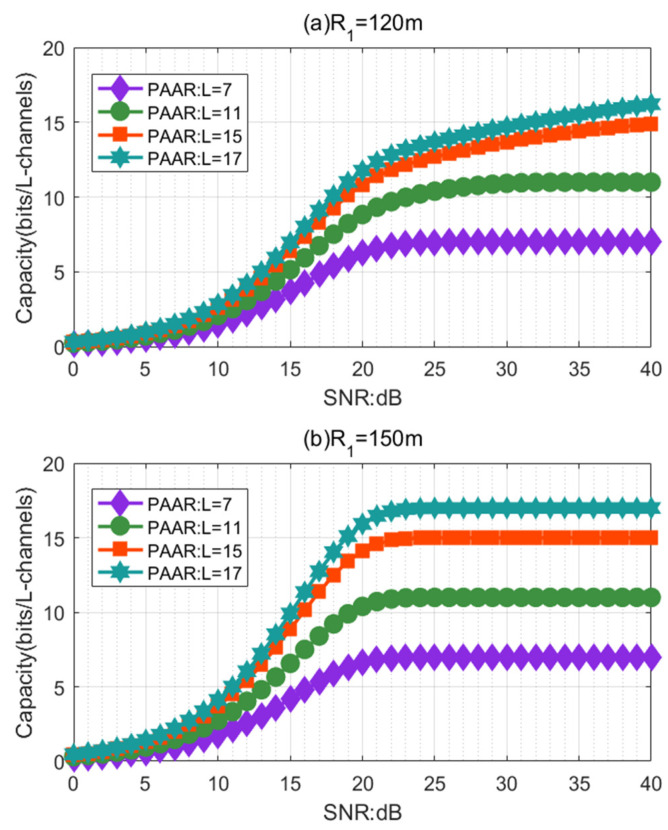
The effect of the SNR on the RV communication system capacity for different radii of the receiver $R_{1}$: (**a**) $R_{1}$ = 120 m; (**b**) $R_{1}$ = 150 m.

Figure 3 delineates the influence of the SNR on the capacity of the RV communication system using the PAAR scheme in the horizontal non-Kolmogorov turbulence environment in the different OAM mode numbers L from 7, 11, 15, to 17. As shown in Figure 3a ($R_{1}$ = 120 m), we can observe that with an identical L, the channel capacity of the PAAR scheme increases with the increase of the SNR. When the SNR is more than 20 dB, the channel capacity of the PAAR scheme gradually increases with the increase of the SNR. Especially for small L values, the growth trend is more gradual. For instance, when *L* is 15 and the SNR is more than 20 dB, the capacity of the PAAR scheme increases 37.44%. However, when the *L* is seven and the SNR is more than 20 dB, the capacity of the PAAR scheme increases only 12.04%. This means that we should choose the appropriate SNR and OAM mode number L to achieve a large capacity. As shown in Figure 3b ($R_{1}$ = 150 m), we can observe that with an identical L, the channel capacity of the PAAR scheme increases with the increase of the SNR. When the SNR is more than 20 dB, the channel capacity of the PAAR scheme gradually reaches the maximum and no longer changes with the increase of the SNR. By comparing Figure 3a,b, it is found that the choice of the receiving aperture leads to different trends in different modes, and in the same situation, increasing the SNR cannot reduce the spread of the beam carrying OAM. At this time, the receiving aperture can be increased to receive more channel capacity.

**Figure 4:**
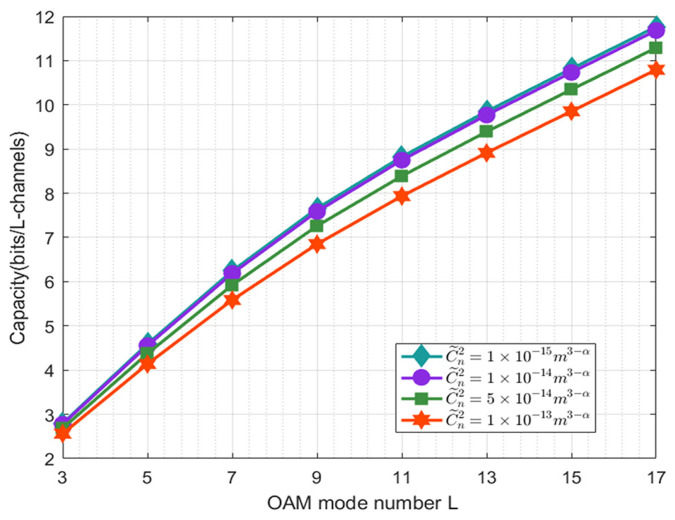
The capacity of the RV communication system against OAM mode number L for different ${\tilde{C}}_{n}^{2}$.

Figure 4 clearly depicts the channel capacity results of varying the number of OAM states for different structure constants (${\tilde{C}}_{n}=1\times 10^{-15}$m${}^{3-\alpha }$, $1\times 10^{-14}$m${}^{3-\alpha }$, $5\times 10^{-14}$m${}^{3-\alpha }$, $1\times 10^{-13}$m${}^{3-\alpha }$) in the horizontal non-Kolmogorov channel model, namely under different turbulence intensities. As shown in Figure 4, we can observe that the channel capacity does not have much difference between ${\tilde{C}}_{n}=1\times 10^{-15}$m${}^{3-\alpha }$ and ${\tilde{C}}_{n}=1\times 10^{-14}$m${}^{3-\alpha }$. However, as the turbulence intensity increases to the moderate and strong levels, the channel capacity of the PAAR scheme decreases, compared to the weak turbulence condition. It is clear that a smaller $C_{n}^{2}$ will cause a larger spatial coherence radius of a spherical wave $\rho_{0}$, and with a larger $\rho_{0}$, we can attribute the improvement in the detection probability to a larger channel capacity.

**Figure 5:**
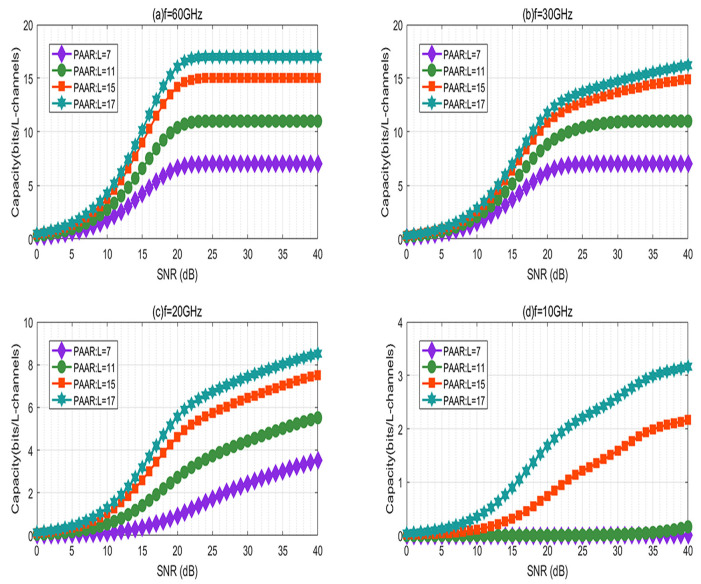
The capacity of the RV communication system against the SNR for different transmission frequencies *f*: (**a**) f = 60 GHz; (**b**) f = 30 GHz; (**c**) f = 20 GHz; (**d**) f = 10 GHz.

In Figure 5, the channel capacity based on the PAAR scheme is simulated as a function of the SNR for different transmission frequencies *f* (f = 60 GHz, 30 GHz, 20 GHz, 10 GHz) under the horizontal non-Kolmogorov channel model. As shown in Figure 5a, we can see that in the case of f = 60 GHz, the channel capacity first increases and then reaches the minimum value as the SNR increases. In Figure 5b,c, we can see that in the case of f = 30 GHz and f = 20 GHz, the channel capacity first increases rapidly, then increases gradually as the SNR increases. As shown by Figure 5d, in the case of f = 10 GHz, L = 15, 17, the channel capacity increases gradually as the SNR increases. However, for L = 7, 11, even if the SNR is increased, the channel capacity is still close to zero, and there is no change. It can be seen from Figure 5 that although increasing the SNR will increase the channel capacity, it is obvious that when f = 60 GHz, the channel capacity is the largest. It is confirmed that in the frequency range selected in this article, a high frequency brings a large capacity of the RV communication system using the PAAR scheme in the horizontal non-Kolmogorov channel model. However, in [25], the capacity of the OAM-mmWave communication systems decreased with the increase of the transmission frequency, where the selected transmission frequency range was 100 GHz–300 GHz. We conclude that the selected transmission frequency range has a great influence on the RV communication system based on the PAAR scheme in a turbulent atmospheric environment.

**Figure 6:**
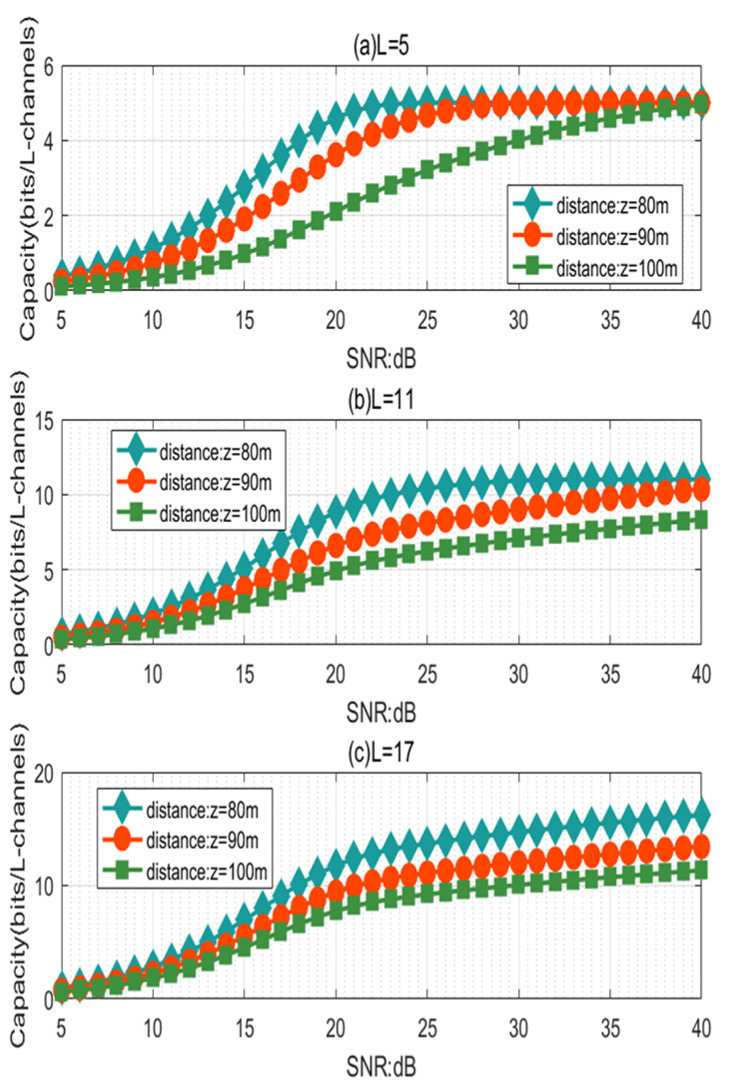
The capacity of the RV communication system against the SNR for different transmission OAM mode numbers L: (**a**) L = 5; (**b**) L = 11; (**c**) L = 17.

Figure 6 illustrates the effect of the SNR on the capacity of the RV communication system using the PAAR scheme for different propagation distances *z* (z = 80 m, 90 m, 100 m) in the horizontal non-Kolmogorov channel model. As shown in Figure 6a, we can observe that in the case of L = 5, with an identical propagation distance *z*, the channel capacity of the PAAR scheme increases with the increase of the SNR. We can also observe that when the SNR increases to 40 dB, the channel capacities are the same under propagation distances z= 80 m, 90 m, 100 m, suggesting that when the OAM mode number L that we transmit is small, we can increase the SNR to increase the propagation distance. As shown in Figure 6b,c (L = 11, 17), we can observe that with an identical propagation distance *z*, the channel capacity of the PAAR scheme increases with the increase of the SNR. When the SNR is more than 20 dB, the channel capacity of the PAAR scheme gradually increases with the increase of the SNR. We can also observe that the channel capacity of the PAAR scheme decreases with the increase of propagation distance *z* with an identical SNR.

**Figure 7:**
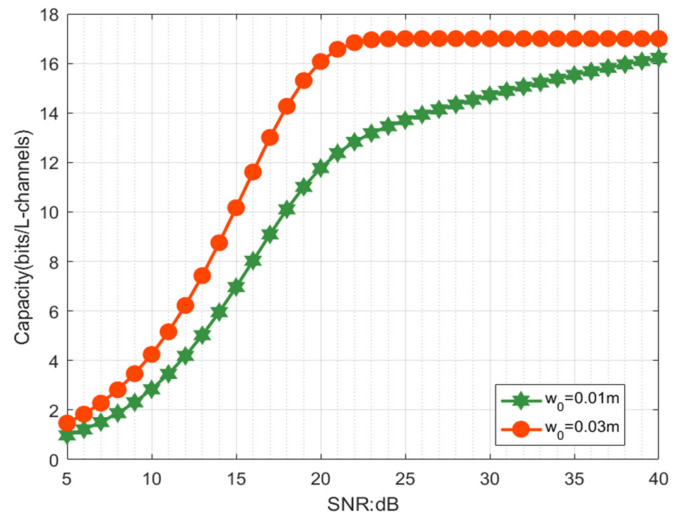
The capacity of the RV communication system against the SNR for different beamwidths $w_{0}$.

Figure 7 explores the impact of the beamwidth $w_{0}$ on the channel capacity of the RV communication system using the PAAR scheme by changing $w_{0}$ = 0.01 m, 0.03 m with different SNRs. We can see that increasing beamwidth $w_{0}$ will lead to a significant improvement of the channel capacity of the PAAR scheme. This is reasonable, since the decrease of $w_{0}$ will result in stronger diffraction and larger mode crosstalk at the receiver.

## 4. Conclusions

In this paper, the influence of atmospheric turbulence on the channel capacity of the RV communication system based on the PAAR scheme is investigated. The spiral spectrum of the PAAR scheme is derived, and we propose the capacity of the RV communication system using the PAAR scheme under the horizontal non-Kolmogorov channel model. Factors affecting the channel capacity, including non-Kolmogorov turbulence parameter α, the signal-to-noise ratio (SNR), structure constant ${\tilde{C}}_{n}^{2}$, transmission frequency *f*, propagation distance *z*, and beam waist radius $w_{0}$, are analyzed in detail. Theoretical analysis and numerical results show that the capacity of the RV communication system using the PAAR scheme is susceptible to turbulence. The capacity of the RV communication system using the PAAR scheme increases with the increase of the SNR and waist radius and with the decrease of the non-Kolmogorov parameter and structure constant. The choice of OAM mode number L has an influence on the propagation distance. When the number of OAM modes L is small, we can improve the SNR to obtain a larger capacity of the RV communication system based on the PAAR scheme over a longer propagation distance. We find that the selected transmission frequency range has a great influence on the RV communication system based on the partial angular aperture receiving scheme. In this paper, the capacity of the RV communication systems increases with the increase of the transmission frequency, where the selected transmission frequency range is 10 GHz–60 GHz. The atmosphere turbulence model is complicated. It is known that the RV communication system’s performance is limited by atmospheric turbulence. In the future, the influence of the atmospheric turbulence model on the performance of the RV communication system based on the PAAR scheme might be further studied. The results acquired in this paper have potential application value in RV communication within non-Kolmogorov turbulence.

## Appendix A. Atmospheric Turbulence Model

### Appendix A.1. Kolmogorov Turbulence Model

For the Kolmogorov turbulence model, the structure function for the index of refraction is:

(A1) $$D_{n}^{Kolmogorov}\left(r\right)=C_{n}^{2}r^{\frac{2}{3}},l_{0}<r<L_{0}$$

where $r=\left|r\right|$ and $C_{n}^{2}$ is the refractive index structure constant [26]. The larger $C_{n}^{2}$ is, the greater the turbulence strength is [23]. $l_{0}$ and $L_{0}$ are the inner and outer scales, respectively. Accordingly, the three-dimensional power spectrum for the index of refraction is [27]:

(A2) $$\Phi_{n}^{Kolmogorov\;}\left(\kappa \right)=0.033C_{n}^{2}\kappa^{-\frac{11}{3}},1/L_{0}\ll \kappa \ll 1/l_{0}$$

where κ is the spatial frequency. The spatial coherence radius of the LG beam under the Kolmogorov turbulence flow model is expressed as:

(A3) $$r_{0}^{Kolmogorov}={\left[\frac{8}{3(\alpha +0.618\Lambda^{\frac{11}{6}})}\right]}^{\frac{3}{5}}{\left(1.46C_{n}^{2}k^{2}z\right)}^{-\frac{3}{5}}$$

with

(A4) $$\alpha =\frac{1-\Theta^{\frac{8}{3}}}{1-\Theta }$$

(A5) $$\Lambda =\frac{2z}{kw^{2}}$$

where $\Theta =1+\frac{z}{R}$ is the curvature parameter of the LG beam at the receiver and Λ is the Fresnel ratio of the LG beam at the receiver.

### Appendix A.2. Non-Kolmogorov Turbulence Model

The index structure function for non-Kolmogorov turbulence is:

(A6) $$D_{n}^{non-Kolmogorov}\left(r\right)=\beta C_{n}^{2}r^{\gamma }.$$

When the exponents γ=2/3, β=1 in the above formula, the above formula evolves into a traditional Kolmogorov turbulent environment structure function of the refractive index. When γ is another value, the units of β are m${}^{-\gamma +2/3}$. When LG beams propagate in a non-Kolmogorov turbulent atmosphere, the corresponding refractive index power spectrum model can be written as [28]:

(A7) $$\begin{array}{cc} \; & \Phi_{n}^{non-Kolmogorov}\left(\kappa \right)=A\left(\alpha \right)C_{n}^{2}\left(z,\alpha \right)\frac{\exp[-\kappa^{2}/\kappa_{m}^{2}]}{{\left(\kappa^{2}+\kappa_{0}^{2}\right)}^{\alpha /2}},0\leq \kappa <\infty ,3<\alpha <5 \end{array}$$

where α is the non-Kolmogorov turbulence parameter, $\kappa_{0}=2\pi /L_{0},\kappa_{m}=c\left(\alpha \right)/l_{0}$, and c(α) is given by:

(A8) $$c\left(\alpha \right)={\left[\Gamma \left(5-\alpha /2\right)A\left(\alpha \right)\frac{2\pi }{3}\right]}^{1/(\alpha -5)}$$

where Γ(x) is the Gamma function. $C_{n}^{2}\left(z,\alpha \right)$ is the generalized refractive index structure constant with units of m${}^{3-\alpha }$ in atmosphere turbulence, which is altitude dependent and given by [19]:

(A9) $$\begin{array}{c} C_{n}^{2}\left(z\cos\theta ,\alpha \right)=0.033{\left(\sqrt{k\cos\theta /h}\right)}^{\alpha -\frac{11}{3}}[0.00594{\left(\nu /27\right)}^{2}\times {\left(h\times 10^{-5}\right)}^{10}exp\left(-h/1000\right)+2.7\times 10^{-16}\times \\ exp\left(-h/1500\right)+C_{n}^{2}\left(0\right)exp\left(-h/100\right)]/A\left(\alpha \right) \end{array}$$

where h=zcosθ is altitude, $C_{n}^{2}\left(0\right)$ is the structure parameter at the ground, ν is the rms wind speed, and θ is the zenith angle [29]. Commonly used values are ν=21 m/s and $C_{n}^{2}\left(0\right)=1.7\times 10^{-14}$m${}^{-\frac{2}{3}}$ in the Hufnagel-Valley model. A(α) is given by:

(A10) $$A\left(\alpha \right)=\Gamma \left(\alpha -1\right)cos\left(\alpha \pi /2\right)/\left(4\pi^{2}\right)$$

$r_{0}$ is the spatial coherence radius of a spherical wave propagating in a non-Kolmogorov channel [27], given by:

(A11) $$\begin{array}{ccc} & r_{0}^{non-Kolmogorov}={\left\{\frac{2\Gamma \left(\frac{3-\alpha }{2}\right){\left[\frac{8}{\alpha -2}\Gamma \left(\frac{2}{\alpha -2}\right)\right]}^{(\alpha -2)/2}}{\pi^{\frac{1}{2}}k^{2}\Gamma \left(\frac{2-\alpha }{2}\right)\int_{0}^{z}C_{n}^{2}\left(\xi ,\alpha \right){\left(1-\xi /z\right)}^{\alpha -2}d\xi }\right\}}^{\frac{1}{\alpha -2}}, & 3<\alpha <4 \end{array}.$$

## Appendix B. The Field Received by the Partial Aperture

According to [9], we know that the aperture is considered a mask. For the whole angular aperture receiving (WAAR) scheme without angle limitation, the mask is:

(A12) M(ϕ)=1,0<ϕ≤2π.

For the WAAR scheme without angle limitation, the received field can be described as E(r,ϕ,z)M(ϕ).

For the PAAR scheme with angle limitation, the mask is:

(A13) $$M^{\prime }\left(\phi \right)=\;\left\{\begin{array}{c} 1,0<\phi \leq \frac{2\pi }{s}\\ 0,\frac{2\pi }{s}<\phi \leq 2\pi \end{array}\right.$$

For the PAAR scheme, since the receiver uses an angular aperture of $\frac{2\pi }{s}$, the received field can be described as $E\left(r,\phi ,z\right)M^{\prime }\left(\phi \right)$. It can be seen that the partial aperture receiving field is $\frac{1}{s}$ of the whole aperture receiving field. s can be defined as the ratio of the received field of the WAAR scheme to the field received by the PAAR scheme. Based on this, we derive Equation (4) in this paper.
